# Gene mutations linked to drug-resistant epilepsy in astrocytoma

**DOI:** 10.3389/fneur.2025.1523468

**Published:** 2025-03-04

**Authors:** Kanitpong Phabphal, Anukoon Kaewborisutsakul, Kittinun Leetanaporn, Pongsakorn Choochuen, Thara Tunthanathip, Raphatphorn Navakanitworakul, Surasak Sangkhathat

**Affiliations:** ^1^Unit of Neurology, Department of Medicine, Faculty of Medicine, Prince of Songkla University, Songkhla, Thailand; ^2^Unit of Neurological Surgery, Department of Surgery, Faculty of Medicine, Prince of Songkla University, Songkhla, Thailand; ^3^Department of Biomedical Sciences and Biomedical Engineering, Prince of Songkla University, Songkhla, Thailand; ^4^Translational Medicine Research Center, Faculty of Medicine, Prince of Songkla University, Songkhla, Thailand; ^5^Department of Surgery, Faculty of Medicine, Prince of Songkla University, Songkhla, Thailand

**Keywords:** drug-resistant epilepsy, receptors, astrocytoma, drug resistance, exome sequencing

## Abstract

**Introduction:**

Epilepsy is common in gliomas, particularly astrocytomas, even in patients who have undergone total tumor resection. Resistance to antiseizure drugs presents a significant challenge in managing epilepsy. Seizure outcomes after brain surgery for drug-resistant epilepsy (DRE) are heterogeneous and difficult to predict using models that evaluate current clinical, imaging, and electrophysiological variables. This study aimed to investigate possible correlations between genetic mutations and antiseizure resistance using whole-exome sequencing.

**Methods:**

Tumor samples from a medical biobank were subjected to whole-exome sequencing, and the contribution of 64 genes from a previous report was analyzed.

**Results:**

Fifteen patients had DRE. Compared to the patients who showed drug responsiveness, patients in the DRE group exhibited mutations in glutamate receptor genes (*GRIA1*, *GRIK5*, *GRIN2B*, or *GRIN2C*), *ATRX*, and the glutamate-S-transferase gene. No significant differences were found between the groups in terms of mutations in *BRAF*, *Olig2*, Ki-67, IDH, PIK3CA, p*53*, *GRM*, or *BCL2A*.

**Discussion:**

These findings suggest that somatic gene mutations are closely linked to DRE. Identifying the molecular basis of antiseizure drug resistance is crucial for improving the management of DRE.

## 1 Introduction

Seizures are among the most frequent clinical manifestations in patients with gliomas. Astrocytomas are one of the most common types of brain tumors, accounting for approximately 75% of all gliomas (1). A recent meta-analysis reported a seizure prevalence of 63–81% in patients with glioma (2). Over half of the patients with glioma experience seizures at least once during the course of their disease (3). The 5-year survival rate of these patients is approximately 70–90% (4). Therefore, achieving better seizure control is crucial for improving the patients’ quality of life (5). However, the risk factors predisposing patients to drug-resistant epilepsy (DRE) remain unclear (6) Recent literature indicates that the histological grading of gliomas fails to capture the complexity of the disease process and does not adequately predict clinical outcomes, especially seizure outcomes (6). Although contemporary studies have reported that some somatic variants are associated with seizure recurrence in gliomas (6, 7), few studies have focused on the somatic variants of DRE in astrocytomas. As long-term survival continues to improve, a better understanding of the natural history and risk factors associated with poor seizure control in adults with astrocytoma is crucial. In recent years, major advances have been made in therapeutic procedures, including new antiepileptic drugs, surgery, chemotherapy, and radiotherapy (8). There remains conflicting evidence, coupled with significant variability in the literature, regarding recurrent seizures and long-term response to antiseizure medication, stratified by brain tumor type. This study aimed to examine long-term seizure control in adults with astrocytoma and identify predictors of DRE.

## 2 Materials and methods

This observational study analyzed the records from the Hospital Information System at Songklanrind Hospital between January 2018 and January 2022. The study included patients with low-grade astrocytoma who presented with seizures and underwent craniotomy with tumor removal. Other treatment modalities, such as radiotherapy and/or chemotherapy, were also evaluated. Patient characteristics, including demographic data, presenting symptoms, neuroimaging findings, data on neurological function, management strategies such as antiseizure medication and steroid use, and type of tumor removal, were collected. Tumor removal was classified as extensive (gross total resection or ≥ 95%) or partial (subtotal resection <95%). Radiological features evaluated included tumor location, size, volume, and peritumoral edema. Tumor size was primarily measured based on the T2/FLAIR hyperintense areas. The assessments of tumor location and characteristics were based on the patients’ medical records and imaging results. The pathological diagnosis was made according to the 2016 WHO guidelines (9). We conducted an extensive review of gene mutations associated with antiseizure medication resistance in the literature and included 71 genes for analysis in this study (Supplementary Table 1).

Epilepsy was diagnostic by neurologist base on the International League Against Epilepsy (ILAE) criteria (10). Refractory epilepsy is defined as the failure of adequate trials of two tolerated and appropriately chosen antiseizure medications (11).

### 2.1 Biological samples and sequencing template preparation

We collected 25 astrocytoma samples from the biobank of the Faculty of Medicine at the Prince of Songkla University. The DNA was extracted using a DNeasy Blood & Tissue Kit (Qiagen, Hilden, Germany), and its quantity and quality were assessed using Nanodrop (Thermo Fisher Scientific, Inc.) and TapeStation (Agilent Technologies, Inc.). The clinical data for all patients were obtained from the hospital’s electronic medical records. Written informed consent was obtained from all patients prior to their recruitment. The study received ethical approval from the Ethical Committee of the Faculty of Medicine at the Prince of Songkla University.

### 2.2 Whole-exome sequencing

Whole-exome sequencing was performed using the Agilent SureSelect XT Human All Exon v8 library preparation system (Agilent Technologies, Inc.). Library quantification was conducted using the Qubit dsDNA High Sensitivity Assay Kit (Invitrogen, Carlsbad, CA, USA), and fragment size was measured using the Agilent D1000 ScreenTape assay. Sequencing was performed on the Illumina NovaSeq-6000 platform (San Diego, California, USA) with paired-end reads of 150 bp. The average targeted coverage depth achieved was 200×. We assessed the quality of the paired-end sequence files using FastQC (version 0.11.9) and trimmed them using Trimmomatic (version 0.39). The BWA program (version 0.7.17) was used to align the FASTQ files to the human reference genome (version GRCh38.13). The resulting Sequence Alignment Map files were converted to Binary Alignment Map files and sorted using SAMtools (version 1.17) (12). The sorted Binary Alignment Map files were regrouped, and duplicate sequences were marked using Picard (version 3.0.0). Base quality score recalibration was performed on non-duplicate Binary Alignment Map files using the Genome Analysis Toolkit (GATK; version 4.4.0). Variant calling was conducted using Mutect2 in the tumor-only mode. A public Panel of Normals was retrieved from the GATK public repository.^1^ We applied GATK4 tools, including GetPileupSummaries, CalculateContamination, and FilterMutectCalls, using the default parameter settings, to filter the variants identified by Mutect2. The identified variants were annotated using Funcotator, and the annotated mutation data were stored in Mutation Annotation Format. We utilized the maftools package in R for the analysis (13).

### 2.3 Statistical analyses

To identify other factors associated with responsiveness to antiseizure medications, we analyzed several genes, grouping the glutamate receptor genes into six groups: (1) alpha-amino-3-hydroxy-5-methyl-4-isoxazolepropionic acid (AMPA) receptors [*GRIA1* (*GluA1*), *GRIA2* (*GluA2*), *GRIA3* (*GluA3*), and *GRIA4* (*GluA4*)]; (2) kainate receptors [*GRIK1* (*GluK1*), *GRIK2* (*GluK2*), *GRIA3* (*GluR3*), *GRIK4* (*GluK4*), and *GRIK5* (*GluK5*)]; (3) NMDA receptors [*GRIN1* (*GluN1*), *GRIN2A* (*GluN2*), *GRIN2B* (*GluN2*), *GRIN3A*, and *GRIN3B*]; (4) [*GRM1* (*mGluR1*), *GRM5* (*mGluR5*); (5) *GRM2* (*mGluR2*), *GRM3* (*mGluR3*); (6) *GRM4* (*mGluR4*), *GRM6* (*mGluR6*), *GRM7* (*mGluR7*), and *GRM8* (*mGluR8*)]. The variables related to the clinical characteristics were expressed as medians [interquartile range (IQR)]. The Mann–Whitney U-test and Fisher’s exact probability test were used to compare the response of patients to antiseizure medication with that of those resistant to it. All statistical analyses were conducted using R version 4.2.1, with a significance threshold of *p*-value.

## 3 Results

A total of 109 patients with low-grade astrocytomas were treated at our institution during the study period. Sixty-two of these patients had preoperative seizures, representing an incidence rate of 56.9%. We excluded data from 37 patients from the analysis: seven patients were lost to follow-up, seven did not undergo immediate postoperative magnetic resonance imaging, and specimens for 23 were not included in the biobank. Thus, 25 patients were included in the study. Of these, 11 were diagnosed with pilocytic astrocytoma, five with pleomorphic xanthoastrocytoma, eight with diffuse astrocytoma, and one with subependymal giant cell astrocytoma. The median time to surgery following seizure onset was 5 months (IQR: 4.8–8.2) in the drug-responsive epilepsy group and 6 months (IQR: 3–8) in the DRE group. At presentation, seizures were focal to bilateral tonic–clonic in 19 patients (76%), focal aware in five patients (20%), and focal with impaired awareness in one patient (4%). At follow-up, 10% (one in 10 patients) of the patients who did not have seizures at presentation developed seizures, which were controlled with antiseizure medication. These patients underwent partial tumor removal and developed recurrent astrocytoma within 14 months.

The median age was 34.6 years (range: 19–51 years). Table 1 summarizes the clinical characteristics of the patients. The median follow-up duration was 34.3 months (IQR: 18.5–39.4 months). All patients received antiseizure medications at admission. Nine patients underwent radiotherapy (six patients with drug-resistance and three who responded to medication). None of the patients had received chemotherapy. The decision to add to therapy in cases of recurrent seizures was based on the physician. Phenytoin was the most commonly used first-line antiseizure medication, prescribed to 15 patients. Levetiracetam was the most commonly used second-line antiseizure medication, followed by valproate. Fifteen patients (60%) had DRE. Of the five patients who experienced perioperative seizures, 15 had recurrent seizures at least five times during follow-up. Eight patients received two antiseizure medications, three patients received three antiseizure medications, and four patients received four antiseizure medications.

**Table 1:** Clinical and pathological characteristics associated with seizure control.

Characteristic	Drug-responsive epilepsy (*n* = 10)	Drug-resistant epilepsy (*n* = 15)	*p*-value
Age (median: IQR; years)	33 (22, 43)	28 (23, 45)	0.93
Sex			0.19
Female	6	12	
Male	4	3	
Tumor histology			0.86
Diffuse astrocytoma	3	5	
Pilocytic astrocytoma	5	6	
Subependymal giant cell astrocytoma	0	1	
Pleomorphic xanthoastrocytoma	2	3	
Duration of seizure before operation (median: IQR; months)	5 (4.8, 8.2)	6 (3, 8)	0.90
Neurological presentation			0.60
Headache	4	8	
Weakness	3	5	
Confusion	2	1	
Other	1	1	
Seizure type before operation			0.70
Focal aware	2	3	
Focal impaired awareness	0	1	
Focal to bilateral tonic–clonic	8	11	
Karnofsky performance scale (median: IQR)	96 (86.8, 95.5)	88 (86, 90)	0.52
Tumor removal			0.61
Extensive	7	9	
Partial	3	6	
Tumor volume (cm^3^) on T2W1	10 (8, 19.3)	11 (7, 19)	0.90
Peritumoral edema	1.7 (0.88, 2.67)	2 (1, 2.4)	0.90
Tumor location			
Temporal	4	8	0.51
Extratemporal	6	7	
Tumor side			0.61
Left	3	6	
Right	7	9	
Steroid used			0.41
Yes	5	5	
No	5	10	
Initial antiseizure medication			0.78
Levetiracetam	1	2	
Valproic acid	2	4	
Phenytoin	7	8	
Lamotrigine	0	1	
Adjuvant therapy			0.61
Radiation only	3	6	
Neither	7	9	
Tumor recurrent			0.70
No	8	11	
Yes	2	4	
Mutations			
*BRAF V600E*	1	2	1.00
*ATRX*	0	7	0.02
*Olig2*	0	1	1.00
Ki-67	0	2	0.50
*PDGFR-a*	0	2	0.50
*PIK3CA*	0	2	0.50
Receptors			
Kainate	0	5	0.04
AMPA	0	2	0.50
NMDA	0	8	0.01
Metabotropic c glutamate	0	3	0.25
Glutamate	0	10	0.01
Glutamate s transferase	0	5	0.01

Kainate: GRIK1, GRIK2, GRIK4, and GRIK5. AMPA: GRIA1. NMDA: GRIN2A, GRIN2C, and GRIN3F. Metabotropic: GRM2, GRM3, GRM5, and GRM6. Glutamate (kainate, AMPA, NMDA, and metabotropic). -, analysis not performed because of the limited number of samples.

**Figure 1:**
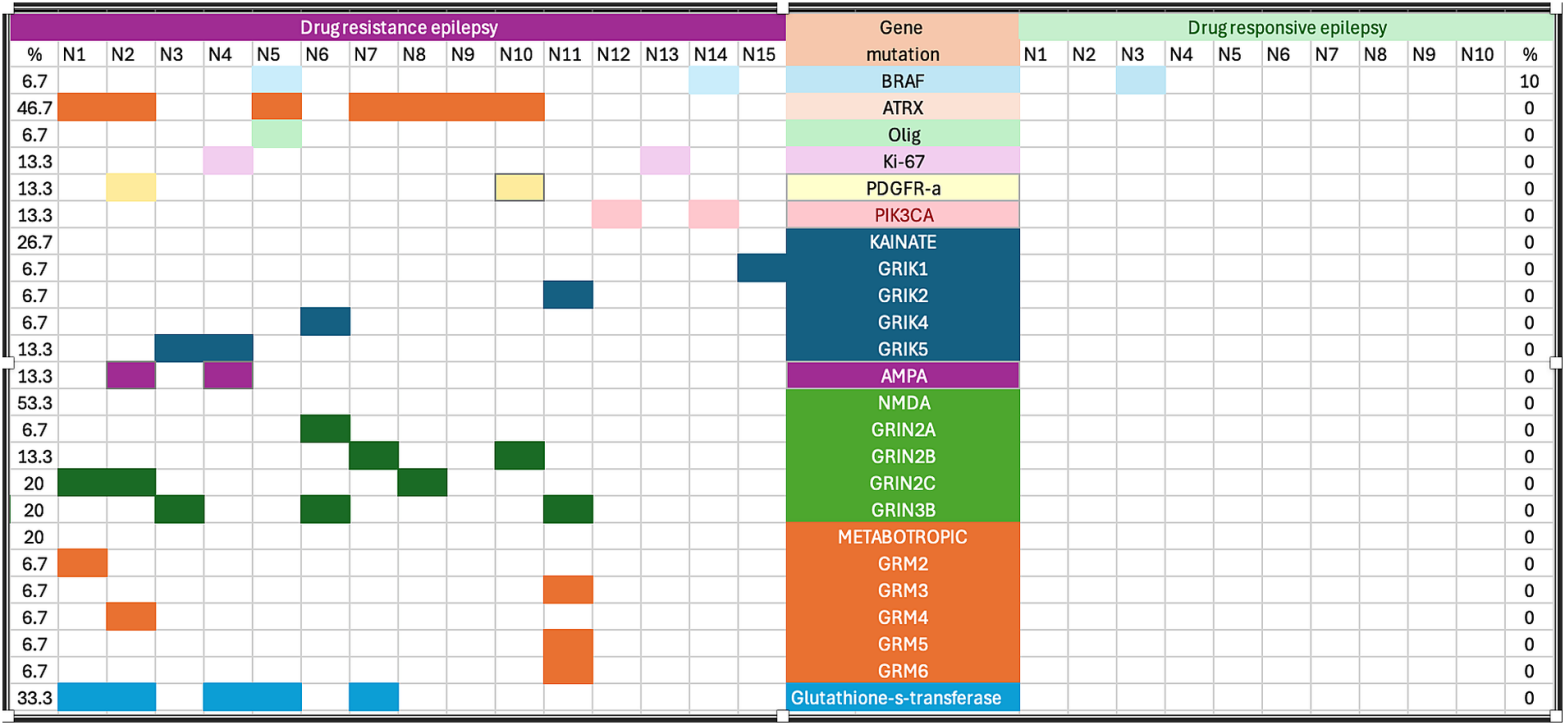
Cohort of 25 paired cases for genetic analysis. AMPA, alpha-amino-3-hydroxy-5-methyl-4-isoxazolepropionic acid.

**Figure 2:**
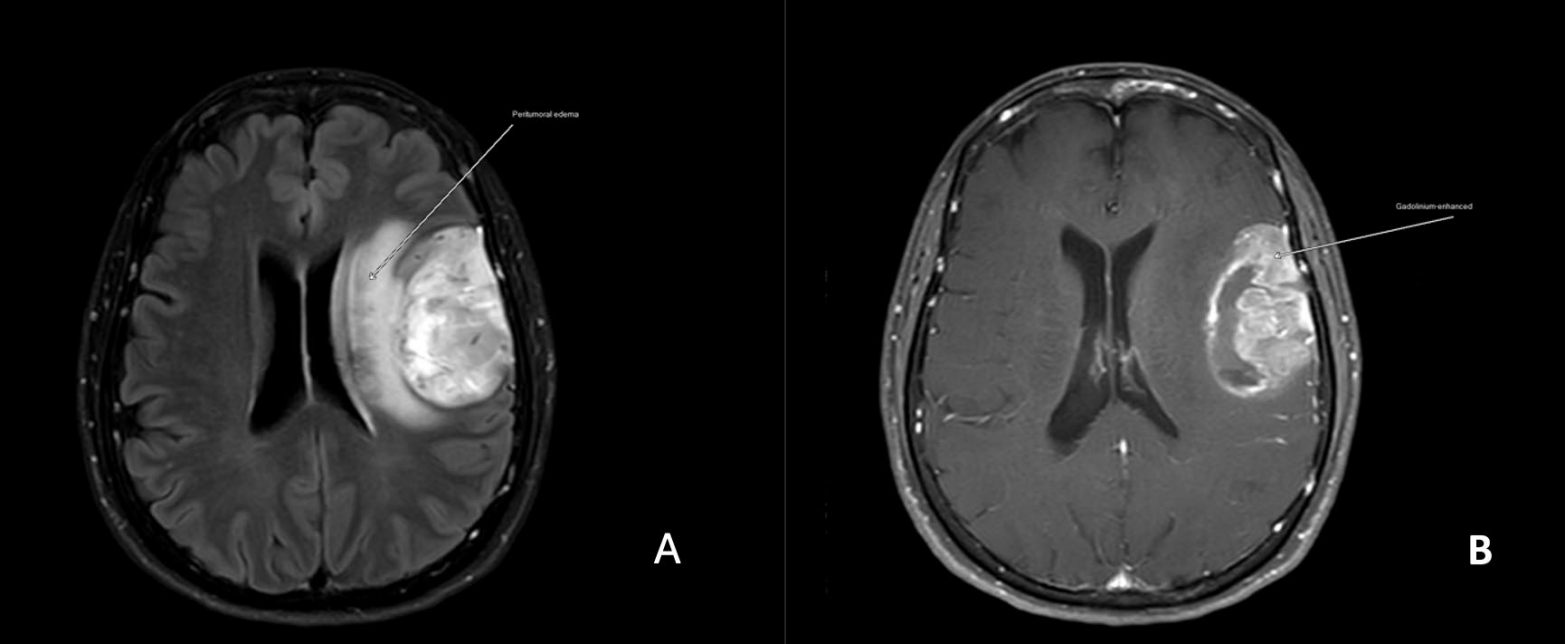
Magnetic resonance images of a patient (N11) with drug-resistant epilepsy and *GRIK2*, *GRIN3B*, *GRM3*, *GRM5*, and *GRM6* mutations **(A)**. The axial 5-mm thick T1-weighted image shows an intra-axial mass at the left frontoparietal operculum with surrounding vasogenic edema (arrow) with heterogeneous enhancement **(B)**.

Three of the 25 patients presented with *BRAF V600E* mutations (one of 10 in the drug-responsive epilepsy group and 2 of 15 in the DRE group). Astrocytomas with the *ATRX* mutation were found in seven of the 15 drug resistance epilepsy samples but were absent in drug-responsive epilepsy. We did not find any association between *ATRX* mutation and histological grading. All patients with *ATRX* mutations had glutamate receptor mutations. Regarding seizure frequency, four patients experienced more than five seizures per month (N1, N2, N6, and N11), and all patients had multiple somatic gene mutations (Figure 1). Other gene mutations detected in our study were *Olig2* (1/25), Ki-67 (2/25), *PDGFR-a* (2/25), *PIK3CA* (2/25), kainate receptor (5/25), AMPA receptor (2/25), NMDA receptor (8/25), metabotropic receptor (3/25), and glutamate transferase (5/25) (Table 1). Figure 2 shows brain magnetic resonance images from a patient with astrocytoma who presented with multiple gene mutations.

Univariate analysis of factors associated with drug-responsive epilepsy and DRE in patients with low-grade astrocytomas showed that compared to those in the former group, patients in the DRE group had glutamate receptor gene mutations (except AMPA), *ATRX*, and glutamate-S-transferase gene mutations (*p* < 0.01) (Table 1). Mutations in the AMPA receptors, *BRAF*, *Olig2*, Ki-67, *PDGFR-α*, *PIK3CA*, *p53*, *BCL2A* were not significantly associated with any differences between the drug-responsive and DRE groups. A table of the specific variants showed in Supplementary Table 1. However, caution is warranted when interpreting these results due to the small sample size (Table 1).

## 4 Discussion

Resistance to antiseizure medication in patients with glioma is a complex multifactorial process. We examined several factors that potentially affect seizure control, including patient characteristics, tumor features, and treatment modalities; however, we did not find any significant association between patient characteristics and DRE. We performed whole-exome sequencing to identify somatic mutations and found that glutamate receptor genes and *ATRX* were associated with DRE.

In our study, the prevalence of seizures in patients with astrocytoma at presentation was 56.9, and 60% of patients developed DRE. Consistent with the results of previous reports, seizures as a presenting symptom of low-grade glioma have been reported in approximately 60–80% of patients (14, 15), with approximately 50% of the patients with low-grade gliomas developing DRE throughout their disease (14, 16).

Traditional factors contributing to brain tumor-associated epilepsy include tumor location, histological type, and malignancy grade. Before the molecular era of brain tumor research, seizures were known to be more common in younger adults and in WHO grade II–III gliomas among patients with diffusely infiltrative gliomas (17). Low-grade astrocytomas are located in the neocortex, where symptoms commonly manifest, with seizures being the most prominent. Previous studies have demonstrated the positive impact of lower-grade gliomas (particularly grade II tumors) on the extent of surgery (3) and radiotherapy or chemotherapy (18) on seizure control. However, other studies have shown that age, sex, neurological symptoms, tumor location, histopathological type, extent of tumor resection, and radiographic features may influence the risk of epilepsy or response to antiseizure medication in patients with glioma. Results from these studies have been conflicting and inconsistent (5, 19, 20). Our results showed no significant association between DRE and factors such as seizure duration before surgery, neurological presentation, Karnofsky Performance Scale score, surgical tumor removal, tumor volume on T2W1, peritumoral edema, tumor location, tumor size, steroid use, or adjunctive therapy with radiation or steroids. These findings reflect the complexity of antiseizure medication resistance, which appears to be influenced by multifaceted pathophysiological mechanisms that a genetic variant might trigger.

The role of genetics in the clinical prediction of neurological disease outcomes is growing, and genetic testing is now widely applied in neurooncology to predict tumor prognosis and treatment responsiveness. However, robust evidence from genetic studies to guide epilepsy treatment decisions is still lacking. While these advancements have improved the ability to predict patient responses to seizure control, the results remain heterogeneous, highlighting the need for further investigation to identify additional clinical predictors. Reports on genetic variations associated with DRE in astrocytomas are limited in the medical literature.

We generated high-depth (>200×) whole-exome sequencing data to identify somatic mutations in low-grade astrocytomas presenting with seizures. We found that mutations in glutamate receptors, *ATRX*, and the glutamate-S-transferase gene were associated with DRE. Mutations in *ATRX* have been identified in multiple tumor types. In 2011, *ATRX* mutations were found to exist in central nervous system tumors, and subsequent studies confirmed that *ATRX* mutations primarily occur in diffuse astrocytomas (21). Additionally, a group of anaplastic astrocytomas with pilocytic features was studied, and *ATRX* loss or pathogenic gene variants were reported in 45% of the cases (22). *ATRX* mutations can be used as prognostic indicators in patients with glioma. Although *ATRX* is a key gene in the development and organization of the brain, its role in epileptogenesis is yet to be fully understood. *ATRX* mutations may indirectly affect the survival and differentiation of inhibitory interneurons, potentially leading to an imbalance between excitatory and inhibitory activity and a predisposition to epilepsy (23). The association between ATRX and DRE in patients with glioma has not been previously described. This is the first study to show that ATRX is associated with DRE in low-grade astrocytomas. However, positive expression of the *ATRX* gene as an independent predictor of preoperative seizures in glioma has been reported (24).

Alterations in growth factor signaling and cell cycle regulation, particularly in the phosphatidylinositol 3-kinase (PI3K)–AKT–mTOR pathway, have been linked to both neoplasia and non-neoplastic cortical malformation syndromes. PIK3CA provides instructions for producing the p110 alpha (p110α) protein, a subunit of the PI3K enzyme. *PIK3CA* mutations affecting the mTOR–MAPK gene network cluster have been associated with focal cortical dysplasia and DRE and other epileptogenic brain lesions (25). *PIK3CA* mutations are common in glioblastoma but rare in low-grade astrocytomas. In our study, we identified *PIK3CA* mutations in only two of the 25 patients with low-grade glioma, both of whom were resistant to antiseizure medications.

Tumors driven by these variants have been associated with key features of epileptogenesis, including selective brain hyperexcitability, synaptic remodeling in tumor cells (26), and peritumoral hyperexcitability. Researchers have established a strong link between the amount of this protein and the cell division cycle. The higher the Ki-67 positivity rate, the greater the proportion of cells in the proliferative cycle, resulting in faster tumor growth. Currently, Ki-67 is an important indicator of tumor cell activity. However, the relationship between Ki-67 and glioma-associated epilepsy in patients with glioma has been underexplored, and existing studies have shown inconsistent results (24). Our results showed that Ki-67 was expressed in three of the 25 patients, all of whom were resistant to antiseizure medication. Although these findings are not conclusive, further research is required to better understand the correlation between the Ki-67 proliferation index and epilepsy in patients with glioma.

Glutamate, a potent excitatory neurotransmitter in the central nervous system, is associated with tumorigenesis in glioma and plays a pivotal role in glioma-associated epilepsy. Evidence shows aberrant glutamate signaling and excessive glutamate function within tumor tissues and their microenvironment. In glioma-adjacent tissues, extracellular glutamate levels were found to be up to 100 times higher than in unaffected brains (27, 28). The release of glutamate into the extracellular space, combined with its reduced reuptake by astrocytes due to the impaired function of excitatory amino acid transporters, contributes to the epileptogenic process. Understanding the role of glutamate in glioma-associated epilepsy is crucial for developing targeted seizure-management therapies (27). Our study found that mutations in glutamate-related genes, including inotropic (kainate and NMDA receptors) and metabotropic (Group 1, Group 2, and Group 3) glutamate receptors, were more frequent in the antiseizure medication resistance group than in the response group. Several approaches have been proposed to modulate glutamate levels, receptors, and transporters to mitigate the excitotoxic effects associated with glioma progression and epileptic activity. Among antiseizure medications, perampanel (PER) is the first-in-class, highly selective, non-competitive, AMPA-type glutamate receptor antagonist (29) and has been identified as potentially beneficial in brain tumor-associated epilepsy. However, data on the use of PER as an adjunct in this patient population is limited. A recent study of 26 patients with uncontrolled seizures due to brain tumor-associated epilepsy treated with PER as an add-on found that eight patients were seizure-free, 15 experienced ≥50% reduction in seizures, and three remained stable. However, molecular profiling was not included in this study. Our study found that only two patients with DRE with astrocytoma had gene mutations in AMPA receptors. Further studies with longer follow-up periods are necessary to confirm this high responder rate and explore the possible correlation between molecular indices and response to PER, which could be a starting point for personalized therapies for patients with brain tumor-associated epilepsy.

Mutations in the isocitrate dehydrogenases (IDH) have been associated with an increased risk of seizures, mainly due to the production of the mutation-2-hydroxyglutarate, which acts as a glutamate analog at excitatory synapses (30). Previous studies have shown that IDH mutations (5, 6) are associated with an increased risk of seizure development, worse postoperative seizure control, and resistance to antiseizure medication in multigrade gliomas (31, 32). A recent meta-analysis suggested that IDH1 and IDH2 mutations are significantly associated with an increased incidence of preoperative seizures in low-grade (II) but not in high-grade (III and IV) gliomas (31). However, IDH mutations are less common in astrocytomas, occurring in only 20% of grade II and 8% of grade III astrocytomas (33). Furthermore, IDH1 or IDH2 mutations have not been identified in any pilocytic astrocytomas (WHO grade I), indicating a different tumorigenic mechanism (32). We did not detect any IDH mutations in our study.

Distortion and deafferentation of cortico-subcortical networks, which may extend beyond the neoplastic lesion, could contribute to seizure persistence and recurrence after gross total tumor resection. The extent of tumor resection is a major prognostic factor and improves seizure control, making surgery a key recommendation in low-grade gliomas (34). In our study, 16 of the 25 patients underwent extensive tumor resection, yet 10 of 15 continued to exhibit antiseizure medication resistance. This suggests that extensive tumor removal may not be sufficient to control seizures in patients with astrocytoma-associated epilepsy.

Our study has several limitations, including a relatively small sample size and individualized treatment approaches, this is the first study to use whole-exome sequencing to identify an association between genetic factors and antiseizure medication resistance over a long-term follow-up. While we performed univariate statistical analysis, future studies should aim to assess the influence of each treatment modality on seizure control. In addition, we did not perform RNA-sequencing or methylation. RNA-sequencing provides a comprehensive characterization of the transcriptome, offering insights into the biological processes and pathways involved in tumorigenesis. It helps in understanding the interactions between tumor cells and their microenvironment, which can inform the development of new therapeutic strategies. Based on this knowledge, we plan to conduct transcriptomics and proteomics studies on a larger population. Finally, we did not perform whole-exome sequencing on peritumoral cortex. The gene expression patterns and mutational landscape of the peritumoral zone differ from those in the glioma core and surrounding brain tissue (35). Additionally, increasing evidence suggests that epileptogenesis is linked to increased and aberrant excitatory synapses observed in the peritumoral area. Changes in the peritumoral cortex, affecting the homeostasis of excitatory and inhibitory neurotransmitters and ionic channel expression, may precede the onset of clinically evident seizures, as shown in animal models (36). Further research is needed to investigate the specific interaction between tumor and normal neurons at the interface and optimize disease.

## 5 Conclusion

We found an association between mutations in glutamate receptor genes, the glutamate-S-transferase gene, and *ATRX* with DRE in patients with low-grade astrocytoma who presented with preoperative seizures. Further studies with a large population of patients with DRE are required to confirm the response rate of antiseizure medication and the possible mechanism of DRE. This study provides a foundation for developing truly personalized therapies for patients with brain tumor-associated epilepsy.

## Data Availability

The datasets generated and/or analysed during the current study are available in the SRA repository [http://www.ncbi.nlm.nih.gov/bioproject/1223041; BioProject ID: PRJNA1223041] case S1-25.

## Footnotes

^1^ https://storage.googleapis.com/gatk-best-practices/somatic-hg38/1000g_pon.hg38.vcf.gz
