# Supplementary material for: Gene mutations linked to drug-resistant epilepsy in astrocytoma
Source: Front Neurol. 2025 Mar 4;16:1523468. doi: 10.3389/fneur.2025.1523468 (PMC11913685; doi:10.3389/fneur.2025.1523468)
Supplement: Supplementary file 1 [file Table_1.DOCX]

| Gene interest |
| --- |
| BRAF, IDH, PIK3CA, FGFR, Forkhead Box O4, VLGR, AQP ,BCL2A, Ki-67, mitogen-activated protein kinase, mTOR, RBBP, PIK3CA, ATRX, TP53, CREBBP, EGFR, KRAS, USP28, cystine-glutamate transporter, secretory carrier membrane proteins, NF, adenosine P, WWOX, TIMP1, PENK, CKAP4, IRS2, MYB, leucine-rich glioma inactivated, adenosine kinase, adenosine deaminase, brain-derived neurotrophic factor, Connexin (Cx)43, Glu, OLIG, RTN, MGMT, platelet-derived growth factor receptor alpha, epidermal growth factor receptor, O(6)-methylguanine DNA methyltransferase, phosphatase and tensin homolog, RAD50 interactor 1, LRP12 promoter, very large G-protein-coupled receptor-1, TERT promoter, miR-128, glyceraldehyde dehydrogenase, neurofilament, GRIA1, GRIA2, GRIA3, GRIA4, GRIK1, GRIK2, GRIA3, GRIK4, GRIK5, GRIN1, GRIN2A, GRIN2B, GRIN3A, GRIN3B, GRM1, GRM5, GRM2, GRM3, GRM4, GRM6, GRM7, GRM8 |

Table 1 gene list glioma-associated seizure
