# Supplementary material for: Gene mutations linked to drug-resistant epilepsy in astrocytoma
Source: Front Neurol. 2025 Mar 4;16:1523468. doi: 10.3389/fneur.2025.1523468 (PMC11913685; doi:10.3389/fneur.2025.1523468)
Supplement: Supplementary file 2 [file Data_Sheet_1.PDF]

Gene	Drug-responsive (N=10)		Drug-resistance (N=15)		p-value
	Sample ID	Variants detected	Sample ID	Variants detected	
BRAF V600E	N3	c.89G>C	N5<br>N14	c.2195C>T<br>c.1780G>A	1.00
ATRX	0	-	N1<br>N2<br>N5<br>N7<br>N8<br>N9<br>N10	c.3616_3619delTCTG<br>c.2968G>T<br>c.4276C>T<br>c.3146delT<br>c.1198G>C<br>c.6547C>A<br>c.4749_4752delGAAA	0.02
Olig2	0	-	N5	c.586G>A	1.00
Ki-67	0	-	N4<br>N13	c.8180G>A<br>c.3461C>G	0.50
PDGFR-a	0	-	N2<br><br>N10	c.1123T>C<br>c.1748A>G<br>c.1369delA<br>c.1372_1409delAATGAAACTTCCTGGACTATTTTGGCCAACAATGTCTC	0.50
PIK3CA	0	-	N12<br>N14	c.91A>C<br>c.331A>G<br>c.1346C>T	0.50
Kainate	0	-	N15<br>N11<br>N6<br>N3<br>N4	GRIK1: c.2152G>A<br>GRIK2: c.875T>C<br>GRIK4: c.2065C>T<br>GRIK5: c.1832T>C<br>GRIK5: c.2917C>A	0.04
AMPA			N2<br>N5	GRIA1: c.622C>T<br>GRIA1: c.2293G>T	0.50
NMDA	0	-	N7<br>N10<br>N6<br><br>N1<br>N2<br>N8<br><br>N3<br>N11	GRIN2B: c.436_437CT>AG<br>GRIN2B: c.785C>T<br>GRIN2A: c.58G>A<br>GRIN3B: c.2424_2425delAA<br>GRIN2C: c.3121C>T<br>GRIN2C: c.2123_2154dup<br>GRIN2C: c.3215C > T,<br>c.4549c>T<br>GRIN3B: c.2349delC<br>GRIN3B: c.1522C>T	0.01
Metabotropic c glutamate	0	-	N1<br>N11	GRM2: c.857A>C<br>GRM3: c.1733T>A	0.25

			N11<br>N11<br>N2	GRM5: c.3173G>C<br>GRM6: c.859C>T<br>GRM4: c.2678G>A	
Glutamate s transferase	0	-	N1<br>N2<br><br>N4<br>N5<br>N7	GSTP1: c.313A>C<br>GSTM1: c.313A>C<br>c.341C>T<br>Arg48Lys: c.143G>A<br>Glu71Lys: c.211G>A<br>Met108Thr:c.323T>C<br>Ala140Asp:c.419C>A	0.01

Supplementary Table 1. All variants annotated in drug-resistant and drug-responsive epilepsy
